# Supplementary material for: A Cross-Sectional Survey of Pediatric Infectious Disease Physicians’ Approach to Congenital Cytomegalovirus Infection
Source: Int J Neonatal Screen. 2023 Mar 24;9(2):17. doi: 10.3390/ijns9020017 (PMC10123618; doi:10.3390/ijns9020017)
Supplement: Supplementary file 1 [file IJNS-09-00017-s001.zip › IJNS-2276334-supplementary.pdf]

Infectious Disease Research Survey

To determine practice patterns for the evaluation and treatment of congenital cytomegalovirus (cCMV). Note that symptomatic cCMV is defined as ≥ 1 visible sign of cCMV, chorioretinitis or intracranial calcifications at the time of birth. Sensorineural hearing loss (SNHL) is not included in this definition and is considered a separate category within the cCMV spectrum of disease (isolated SNHL). **Correct answers highlighted in yellow.**

* Required

1. How old are you? [years]

a. Your answer

2. Gender

a. Male

b. Female

c. Prefer not to say

c. Other

4. Years in Practice

a. Still in training

b. 0-5 years

c. 6-10 years

d. 11-15 years

e. More than 15 years

5. Race/Ethnicity (Check all that apply)

a. American Indian or Alaska Native

b. Asian

c. Black or African American

d. Hispanic

e. Native Hawaiian or Pacific Islander

f. White

g. Other (Please specify) ________________

6. Please choose the best answer that describes your position.

a. Pediatric Infectious Disease, Fellowship Trained community practice
b. Pediatric Infectious Disease, Fellowship Trained academic practice
c. Pediatric Infectious Disease Fellow, In training
d. Other: (Please specify) ___________________

7. What percent of medicaid patients comprise your practice ? [%]

_______________________(short answer text)

May check more than one option
a. Academic or University Private Practice
b. Managed care
c. Hospital setting
d. Other: (Please specify)_____________________

8. What percent of medicaid patients comprise your practice? ____ %

Fund of Knowledge Questions. I apologize if these questions are overly simple. They will be used to compare your response to other subspecialties.

9. What symptoms/signs are associated with cCMV infection? (Pick all that apply)
a. hearing loss
b. intellectual disability
c. loss of vision
d. seizures
e. oral cavity ulcers
f. petechia

Reference: Goderis J et al. Hearing Loss and Congenital CMV Infection: A Systematic Review. Pediatrics. 2014.

11. Which of the following statement(s) regarding congenital cytomegalovirus (cCMV) is/are true? (Pick all that apply)

a. Approximately 10% of children with asymptomatic cCMV will develop sensorineural hearing loss (SNHL).
b. Approximately 30% of children with asymptomatic cCMV will develop SNHL.
c. Approximately 33% of children with symptomatic cCMV will develop SNHL.
d. Approximately 95% children with symptomatic cCMV will develop SNHL.

Reference: Goderis J et al. Hearing Loss and Congenital CMV Infection: A Systematic Review. Pediatrics. 2014.

12. Of children with cCMV associated SNHL, how many will develop progressively worsening SNHL?
a. 5%
b. 20%
c. 35%
d. 50%

Reference: Goderis J et al. Hearing Loss and Congenital CMV Infection: A Systematic Review. Pediatrics. 2014.

13. What test(s) should be performed to diagnose cCMV? (pick all that apply)
a. Dried blood spot CMV PCR after 3 weeks of age.
b. Dried blood spot CMV PCR prior to 3 weeks of age.
c. Urine PCR/culture at any age
d. Urine PCR/culture prior to 3 weeks of age.
e. Saliva PCR/culture at any age
f. Saliva PCR/culture prior to 3 weeks of age
g. Saliva CMV culture with confirmatory urine PCR or culture at any age.
h. Saliva CMV culture with confirmatory urine PCR or culture prior to 3 weeks of age.
i. Serologic CMV IgG testing at any age.
j. Serologic CMV IgG testing prior to 3 weeks of age

Responses are based on the time sensitive nature of cCMV diagnosis and sensitivity of the various assays. Reference: Rawlinson WD et al. Congenital Cytomegalovirus infection in

pregnancy and the neonate: consensus recommendations for prevention, diagnosis and therapy. The Lancet, 2017.

14. Which of the following activities could result in CMV transmission (pick all that apply).

a. Kissing

b. Changing diapers without hand washing afterwards

c. Drinking breast milk

d. Receiving a blood transfusion

e. Sexual intercourse

f. Sharing food

Reference: Rawlinson WD et al. Congenital Cytomegalovirus infection in pregnancy and the neonate: consensus recommendations for prevention, diagnosis and therapy. The Lancet, 2017.

15. Which test(s) can definitively establish a diagnosis for cCMV in children greater than 3 weeks of age? (Select all that apply)

a. Dried blood spot CMV PCR testing

b. Serology for CMV IgG

c. Serology for CMV IgM

d. Imaging studies including CT and MRI

e. Urine PCR/culture for CMV

f. Saliva culture for CMV

Reference: Boppana SB et al. Dried Blood Spot Real-time Polymerase Chain reaction Assays to Screen Newborns for Congenital Cytomegalovirus Infection. JAMA. 2010.

16. A child with SNHL undergoes dried blood spot CMV PCR testing. The results show CMV is undetectable. How do you counsel the family?

a. cCMV cannot be ruled out as an etiology for the child's SNHL.

b. cCMV can be ruled out as an etiology for the child's SNHL.

Reference: Boppana SB et al. Dried Blood Spot Real-time Polymerase Chain reaction Assays to Screen Newborns for Congenital Cytomegalovirus Infection. JAMA. 2010.

CMV testing and treatment:

17. What is your institutional protocol for early CMV testing/screening? (pick all indications that apply)?

a. All infants
b. Any infant in the Neonatal Intensive Care Unit
c. Documented maternal CMV infection
d. Failed newborn hearing screening
e. Hepatosplenomegaly
f. Microcephaly
g. No institutional protocol
h. Petechial rash
i. Small for gestational age (SGA)
j. SNHL
k. Unexplained thrombocytopenia
l. Other (Please specify) ____________________________

18. In addition to your institutional protocol, what other indications prompt you personally to screen//test for cCMV?

a. All infants
b. Any infant in the Neonatal Intensive Care Unit
c. Documented maternal CMV infection
d. Failed newborn hearing screening
e. Hepatosplenomegaly
f. Microcephaly
g. None
h. Petechial rash
i. SGA
j. SNHL
k. Unexplained thrombocytopenia
l. Other (Please specify) ____________________________

19. For infants recently diagnosed with cCMV infection, which of the following do you recommend? (Pick all that apply) Neuroimaging will be asked in the following question.

a. No additional testing
b. CBC with differential
c. Comprehensive metabolic profile
d. Ophthalmology evaluation
e. Diagnostic hearing testing
f. Developmental evaluation
g. Early intervention referral
h. Other (Please specify) ____________________________________

20. If you order neuroimaging, which of the following responses most closely follow your clinical practice for an infant recently diagnosed with cCMV? (Pick all that apply)

a. Head ultrasound (HUS) for all cases
b. Brain MRI for all cases
c. HUS for all cases followed by a brain MRI only if an abnormality if found
d. HUS except a Brain MRI if infant presents with microcephaly
e. Other (Please specify) ____________________________________

21. In your practice, what are your indications to recommend antiviral therapy in a cCMV infected infant (Valganciclovir)? (Pick all that apply)

a. < 1 mo old newborn with no signs or symptoms associated with cCMV infection.
b. Between 1-6 month old infant with no signs or symptoms associated with cCMV infection.
c. Older than 6 month old infant with no signs or symptoms associated with cCMV infection.
d. < 1 mo old newborn with isolated sensorineural hearing loss (SNHL).
e. Between 1-6 month old infant with isolated sensorineural hearing loss (SNHL).
f. Older than 6 month old infant with isolated sensorineural hearing loss (SNHL).
g. < 1 mo old newborn with one of the following: SGA, microcephaly, thombocytopenia, or petechial rash.
h. Between 1-6 month old infant with one of the following: SGA, microcephaly, thombocytopenia, or petechial rash.
i. Older than 6 month old infant with one of the following: SGA, microcephaly, thombocytopenia, or petechial rash.

j. < 1 mo old newborn with 2 of the following: SNHL, isolated SGA, microcephaly, thombocytopenia, or petechial rash.

k. Between 1-6 month old infant with 2 of the following: SNHL, isolated SGA, microcephaly, thombocytopenia, or petechial rash.
l. Older than 6 month old infant with 2 of the following: SNHL, isolated SGA, microcephaly, thombocytopenia, or petechial rash.

m. Other indication (s): ______________________________________

17. How frequently do you obtain absolute neutrophil count values for patients on Valganciclovir treatment?
a. Weekly
b. Every other week
c. Monthly
d. Not at all
e. Weekly for 6 weeks then every other week for 1 month then monthly until treatment completed
f. Other (please specify schedule): ______________________________________

18. Indicate any other tests you routinely order when administering Valganciclovir for an infant with cCMV.
a. CBC with differential
b. Viral titers (e.g. urine CMV PCR quantitative)
c. Drug concentration or phamacokinetic studies.
d. Comprehensive metabolic profile levels (e.g. liver function testing)
e. Drug resistance
f. Other (Please specify): __________________________

19. How long do you administer valganciclovir?
a. 6 weeks
b. 6 months
c. 9 months
d. 12 months
e. Until urine viral titers are undetectable.
f. Other (Please specify): ___________________

20. How often so you recommend follow up hearing testing for an infant with cCMV?

a. Every 3 months for 3 years then every 6 months until 6 years of age then annually afterwards

b. Every 3- 6 months in the first year then every 6 months until 3 years of age then every 12 months until 6 years of age.

c. Every 6 months for the first 3 years then annually thereafter through adolescence

d. Other (Please specify) ___________________

21. What do you consider “treatment” for cCMV? (Pick all that apply)

a. Valganciclovir administration

b. Serial hearing testing

c. Early intervention

d. Hearing aids or cochlear implantation if indicated

e. Other (Please specify): _______________________

22. We appreciate any comments or suggestions ___________________________________
